# Specificity of Transmembrane Protein Palmitoylation in Yeast

**DOI:** 10.1371/journal.pone.0016969

**Published:** 2011-02-24

**Authors:** Ayelén González Montoro, Sabrina Chumpen Ramirez, Rodrigo Quiroga, Javier Valdez Taubas

**Affiliations:** Centro de Investigaciones en Química Biológica de Córdoba, CIQUIBIC (UNC-CONICET), Departamento de Química Biológica, Facultad de Ciencias Químicas, Universidad Nacional de Córdoba, Córdoba, Argentina; Institute of Developmental Biology and Cancer Research, France

## Abstract

Many proteins are modified after their synthesis, by the addition of a lipid molecule to one or more cysteine residues, through a thioester bond. This modification is called S-acylation, and more commonly palmitoylation. This reaction is carried out by a family of enzymes, called palmitoyltransferases (PATs), characterized by the presence of a conserved 50- aminoacids domain called “Asp-His-His-Cys- Cysteine Rich Domain” (DHHC-CRD). There are 7 members of this family in the yeast *Saccharomyces cerevisiae,* and each of these proteins is thought to be responsible for the palmitoylation of a subset of substrates. Substrate specificity of PATs, however, is not yet fully understood. Several yeast PATs seem to have overlapping specificity, and it has been proposed that the machinery responsible for palmitoylating peripheral membrane proteins in mammalian cells, lacks specificity altogether.

Here we investigate the specificity of transmembrane protein palmitoylation in *S. cerevisiae*, which is carried out predominantly by two PATs, Swf1 and Pfa4. We show that palmitoylation of transmembrane substrates requires dedicated PATs, since other yeast PATs are mostly unable to perform Swf1 or Pfa4 functions, even when overexpressed. Furthermore, we find that Swf1 is highly specific for its substrates, as it is unable to substitute for other PATs. To identify where Swf1 specificity lies, we carried out a bioinformatics survey to identify amino acids responsible for the determination of specificity or **S**pecificity **D**etermination **P**ositions (SDPs) and showed experimentally, that mutation of the two best SDP candidates, A145 and K148, results in complete and partial loss of function, respectively. These residues are located within the conserved catalytic DHHC domain suggesting that it could also be involved in the determination of specificity. Finally, we show that modifying the position of the cysteines in Tlg1, a Swf1 substrate, results in lack of palmitoylation, as expected for a highly specific enzymatic reaction.

## Introduction

Protein palmitoylation or S-acylation is the addition of a lipid molecule on a cysteine residue of a protein by thioestherification. This is the only lipid modification that is reversible and thus susceptible to regulation [1], [2], [3]. This modification is of great relevance in the regulation of several important processes such as the visual cycle [4], signal transduction [5], and synaptic transmission [6], [7], [8]. For instance, palmitoylation regulates localization and signalling activity of Ras isoforms, and hence diverse signalling pathways [9]. Many proteins are palmitoylated in neurons, where this modification modulates sorting of presynaptic proteins, synapse morphology and clustering of ion channels [7], [8], [10].

A family of proteins containing a 50 residues long domain called Asp-His-His-Cys Cysteine Rich Domain (DHHC-CRD) [11] is involved in protein S-acylation [12], [13], reviewed in [14]. There are at least 23 predicted DHHC-CRD containing proteins in the human genome and 7 in the *Saccharomyces cerevisiae* genome. They are integral membrane proteins predicted to contain 4 to 6 transmembrane domains. Subsets of substrates have been assigned to most of the yeast palmitoyltransferases (PATs) [12], [13], [15], [16], [17] and to several mammalian PATs [18], [19], [20].

Palmitoyltransferases make interesting drug targets for therapeutic intervention, mainly because of their involvement in the modification of oncoproteins [21] and their role in modulating neuronal trafficking and function [6], [7], [9]. However, a deeper understanding of palmitoylation and its consequences is required.

Recent progress in the field has consisted mostly in the identification of PATs and their substrates. Basic knowledge regarding the mechanism, the enzymes responsible for this modification and their regulation is lacking. Few residues have been mutated resulting in lack of function [12], [13], [17], [22], [23], and most are highly conserved residues located within the DHHC domain. Outside this domain, a mutation at position 3 of the recently identified PaCCT (Palmitoyltransferase Conserved C-Terminus) motif, present in most PAT’s C-termini, results in lack of function for at least two yeast PATs, Swf1 and Pfa3 [24].

An aspect that requires further investigation is the specificity of different PATs towards their substrates, and how it is determined. Based on sequence comparison, it has been assumed that since the DHHC domain is highly conserved in the family, it represents the catalytic core, while specificity would be determined within the highly variable N- and C-termini [14], [24].

Although there are no consensus sequences identified in palmitoylation substrates, a few determinants necessary for recognition by PATs have been described [25], [26]. Nevertheless, several substrates can be palmitoylated by more than one PAT. Recently, Hou et al, (2009) [23], have shown that phenotypes associated with the lack of Pfa3, a yeast PAT responsible for the palmitoylation of the vacuole fusion factor Vac8 [17], can be suppressed upon high overexpression of most yeast PATs, leading to postulate that some PATs have overlapping specificity. Accordingly, *in vivo* palmitoylation of Vac8 is only partially reduced in the absence of Pfa3 [17]. Also, Ras2 palmitoylation is only partially suppressed in the absence of its cognate PAT, Erf2 [15], [27]. Neuronal PATs exhibit distinct substrate specificity, although most substrates studied are modified by more than one PAT [18], [28]. Overlapping specificity for some yeast PATs was also shown using an elegant proteomics approach [15]. More recently, it has been postulated that in mammalian cells, the palmitoylation machinery for peripheral membrane proteins is devoid of specificity [29].

Swf1 is a yeast DHHC-protein involved in the palmitoylation of SNARE (Soluble N-ethylmaleimide-sensitive factor attachment protein Receptor) fusion proteins [16] and possibly glycosyltransferases [15]. These represent a set of substrates that consist of type II transmembrane proteins that are palmitoylated in cysteines adjacent to the cytosolic border of the transmembrane domain (TMD). The function of transmembrane SNARE palmitoylation is not clear, but in the case of the endosomal syntaxin Tlg1, it seems to protect it from degradation by the quality control machinery [16].

Palmitoylation of multi-spanning membrane proteins in yeast appears to be carried out mostly by Pfa4. This protein palmitoylates several amino acid permeases [15] and the chitin synthase Chs3, allowing it to exit the ER [30].

In this work we investigate the specificity of transmembrane protein palmitoylation mediated by Swf1 and Pfa4, and also where is Swf1 specificity encoded.

## Results

### Swf1 is unable to substitute for other PATs

Swf1 mediates the palmitoylation of type II membrane proteins such as SNAREs and possibly glycosyltransferases [15], [16]. We were interested in the specificity of this modification, particularly in the light of two recent studies, one by Rocks et al. (2010), in mammalian cells, where they postulate that palmitoylation of peripheral membrane proteins lacks specificity, and another by Hou et al. (2009), where they show that overexpression of Erf2, Pfa4, or Akr1 is able to partially suppress phenotypes associated with lack of PFA3 [23].

**Figure 1:**
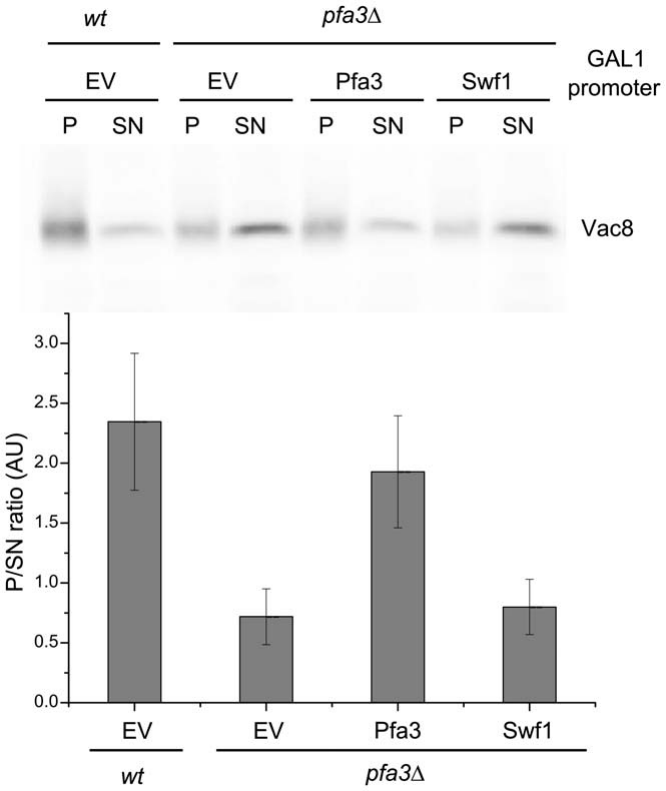
Swf1 does not complement a *pfa3*Δ strain. Western blot analysis of Vac8 membrane/cytosol distribution in a *wt* or *pfa3*Δ strains transformed with an empty vector (EV*)* or expressing GAL1-promoter driven Pfa3-PA or Swf1-PA. The blot was developed using anti-Vac8 antibodies. The bands from four independent experiments were quantified and the ratio between the amount of Vac8 present in the the pellet corresponding to the membrane fraction (P) and the supernatant (SN) was plotted in a bar graph (lower panel).

Vac8 is a protein involved in vacuolar fusion which, when palmitoylated, localizes to the vacuolar membrane [31]. We analyzed the distribution of Vac8 by Western blots of membrane vs. soluble fractions for a *wt* strain, a *pfa3*Δ strain and a *pfa3*Δ strain overexpressing Swf1 from a GAL1 promoter. Figure 1 shows that overexpression of Swf1 does not alter the distribution of Vac8 in a *pfa3*Δ strain. GAL1 promoter driven expression of Swf1 is also unable to complement a *pfa4*Δ strain (see below), indicating that Swf1 is less promiscuous than other PATs.

### Swf1 and Pfa4 activities are not carried out by other yeast PATs

We next analyzed whether other PATs can complement Swf1 function. This can be evaluated by growth tests on media containing lactate as the sole carbon source, in which *swf1*Δ cells cannot grow. The bases for this phenotype are unknown, but it is a good predictor of Swf1 palmitoylation activitity. A *swf1*Δ *tul1*Δ strain in which Tlg1 is not degraded still does not grow in lactate indicating that the degradation of Tlg1 is not responsible for this phenotype. [16], [24] Moreover, we have screened the whole collection of yeast deletion mutants for strains that show growth defects in both lactate and high salt, another phenotype of the *swf1*Δ strain, We identified *swf1*Δ in this screen, as expected, but none of its cognate substrates, suggesting that this phenotype cannot be assigned to lack of function of any particular known substrate (Gonzalez Montoro and Valdez Taubas, unpublished).

**Figure 2:**
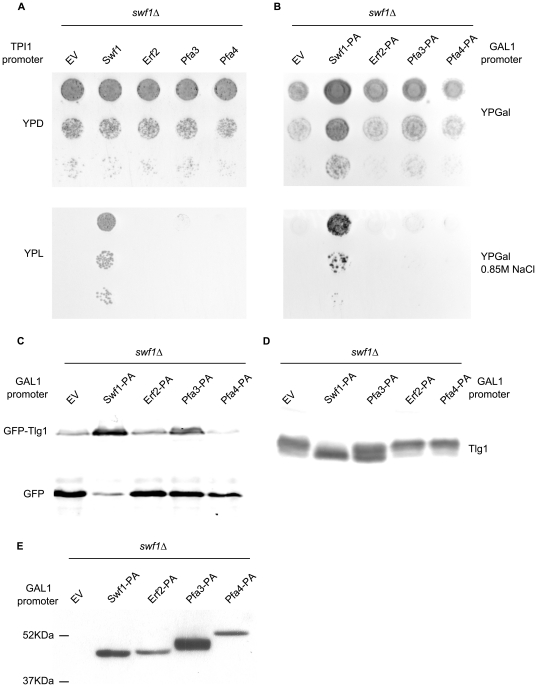
Swf1 substrates modification by other PATs. **A**. Serial dilutions of a *wt* strain or *swf1*Δ strains transformed with empty vector (EV), or plasmids containing untagged Swf1, Erf2, Pfa3 or Pfa4 under the control of the TPI1 promoter. Transformants were grown in solid rich medium containing glucose (YPD, upper panel) or in solid rich medium containing lactate as the sole carbon source (YPL, lower panel). **B**. Serial dilutions of a *wt* strain or a *swf1*Δ strain transformed with an empty vector (EV), or with plasmids containing protein-A tagged Swf1, Erf2, Pfa3 or Pfa4 under the control of the inducible GAL1 promoter. Transformants were grown in solid rich medium containing galactose as the sole carbon source (YPGal, upper panel) or in medium supplemented with 0.85 M NaCl (YPGal 0.85 M NaCl). **C**. Western blot analysis to assess the degradation of GFP-Tlg1 in *swf1*Δ strains overexpressing three different yeast PATs. The blot was developed using anti-GFP antibodies. The amount of free GFP label is indicative of GFP-Tlg1 sorting to the vacuoles, an indirect measure of its palmitoylation status. **D**. The palmitoylation status of Tlg1 was analyzed in a biotinylation assay (see Materials and methods). Membrane proteins from a *swf1*Δ strain transformed with an empty vector (EV), or with GAL1 promoter driven Pfa3, Erf1, Pfa4 or Swf1, were treated with biotin-BMCC and subjected to Western blot using anti-Tlg1 antibodies. **E**. Western blot analysis of the different PATs expression levels when driven by the inducible Gal1 promoter. The proteins are tagged at the C-terminus with the IgG binding domain of protein A (PA).

**Figure 3:**
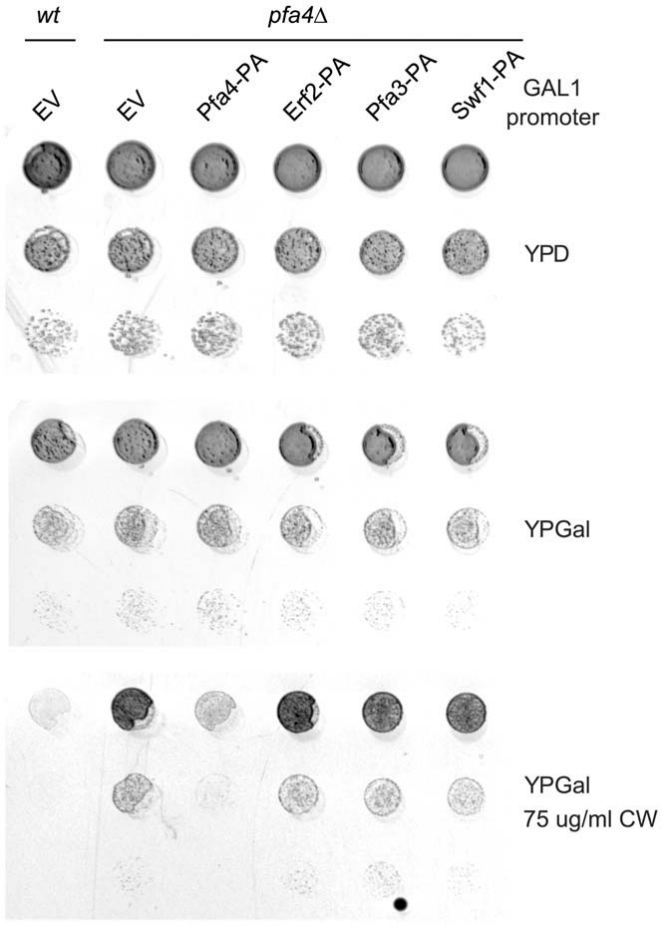
Pfa4 function cannot be carried out by other yeast PATs. Serial dilutions of a *wt* strain or a *pfa4*Δ strain transformed with an empty vector (EV), or plasmids containing protein-A tagged Swf1, Erf2, Pfa3 or Pfa4 under the control of the GAL1 promoter. Transformants were grown in solid rich medium containing glucose (YPD, upper panel) or galactose (YPGal, medium panel) as carbon source, or in YPGal supplemented with 75 µg/mL Calcofluor White (YPGal CW, lower panel).

Figure 2A shows that, when driven from a TPI1 promoter, which normally leads to moderate overexpression of proteins, neither Erf2, Pfa3 nor Pfa4 can complement *swf1*Δ growth deficiency in media containing lactate as a carbon source. To make our experiments comparable to those of Hou et al (2009), we expressed these constructs under the strong GAL1 promoter, and analyzed the phenotypes of *swf1*Δ strains. In this case, galactose must be present in the media, so we evaluated growth in the presence of a high concentration of NaCl. *swf1*Δ is unable to grow in 0.85M NaCl [16], [32] a feature it shares with other VPS mutants [32]. Since galactose is a poor carbon source, *swf1*Δ growth deficiency could already be observed without salt addition (Figure 2B upper panel). With high salt, the PATs tested did not complement *swf1*Δ and we only see a very limited growth in the presence of overexpressed Pfa3 (Figure 2B, lower panel). We confirmed these results by analyzing the vacuolar degradation of the Swf1 substrate Tlg1, which can be directly correlated to its palmitoylation status [16], [24]. In a Western blot analysis of a *swf1*Δ strain expressing a GFP-Tlg1 fusion, some of the label will be in the form of free GFP which is resistant to vacuolar proteolysis. Figure 2C shows that GAL1 driven PATs are unable to fully suppress GFP-Tlg1 degradation. A small amount of GFP-Tlg1 is protected from degradation upon Pfa3 overexpression, in agreement to what is observed in growth test experiments. Finally, we assessed the palmitoylation status of Tlg1 directly. Instead of the classical biotinyl exchange, we carried out direct biotinylation experiments, since Tlg1 has only two cysteines and they are both modified by palmitate *in vivo* [16]. The results can be observed by analyzing the shift towards higher molecular weights that is produced in the proteins by the reaction with biotin-BMCC. This shift does not occur when the cysteines are protected by palmitoylation. Figure 2D shows that, in a *swf1*Δ strain, Tlg1 shifts towards higher molecular weights compared to the same strain complemented with wt Swf1. When the *swf1*Δ strain was transformed with GAL1 promoter driven Erf2 or Pfa4, Tlg1 behaves as in the empty vector (EV) control, indicating that these PATs are unable to palmitoylate Tlg1 even when overexpressed. Again, when we analyze the effect of Pfa3, an intermediate situation is observed. Figure 2E shows the expression levels of GAL1-driven PATs, tagged at the C-terminus with the IgG binding domain of Protein A. The functionality of all these constructs was tested in complementation assays of the corresponding deletion mutants: Figure 1 for Pfa3, Figure 2 for Swf1, and Figure 3 for Pfa4. Erf2-PA complementation assay is not shown. Similar Protein-A tagged constructs were previously proven to be functional [23]. These results are consistent with those obtained with TPI1 driven constructs, which are untagged.

These results show directly that palmitoylation of the Swf1 substrate Tlg1 cannot be carried out by other PATs, and confirm that, *in vivo*, this activity is exclusively performed by Swf1, because only upon massive overexpression of Pfa3 a small degree of complementation is observed. It should be noted that Pfa3 is over-expressed at much higher levels than the other PATs (Figure 2E). The growth tests strongly suggest that this is also valid to at least another, or possibly several Swf1 substrates.

We extended our observations to another PAT, Pfa4, which is responsible for the palmitoylation of polytopic membrane proteins such as aminoacid permeases and chitin synthase 3 (Chs3) [15], [30]. One of the phenotypes of a *pfa4*Δ strain is its ability to grow in the presence of Calcofluor White (CW). This compound binds to chitin present in the yeast cell wall. *pfa4*Δ cells have reduced chitin levels and are therefore resistant to 75 µg/ml CW, while wt cells cannot grow in these conditions [30]. This is due to unpalmitoylated Chs3 being trapped in the ER in the absence of Pfa4, which precludes its function in chitin synthesis [30]. This allows us to evaluate the function of Pfa4, at least regarding Chs3 palmitoylation, in simple growth tests in the presence of CW.

We transformed a *pfa4*Δ strain with GAL1 promoter driven Swf1, Pfa3, and Erf2. Figure 3 shows that neither of these constructs is able to suppress the CW phenotype of a *pfa4*Δ mutant, resulting in CW resistant strains. These experiments show that Pfa4 activity towards CHS3 cannot be carried out by other PATs and suggest that palmitoylation of Pfa4 substrates is also highly specific.

### In silico analysis predicts the presence of Specificity Determination Positions within the DHHC domain of PATs

Domains or motifs that mediate Swf1 or Pfa4 specificity are not obvious to identify by sequence comparison, since even orthologues from closely related organisms display low conservation outside the DHHC region, the TTxE motif and the PaCCT motif [24].

We used an alternative approach based on the prediction of Specificity Determination Positions (SDPs). Within a given family of proteins, members can be grouped according to substrate specificity. Often, differences in specificity between the groups can be ascribed to single residues that are conserved within the members of the subgroup and not in the members of other specificity groups [33]. These positions are known as specificity determination positions. Bioinformatic tools have been designed to identify these SDPs. We used GROUPSIM [34], which considers residue conservation within specificity groups and physicochemical characteristics of the residues.

It should be stated that SDPs do not necessarily refer to specificity in terms of biochemical affinity for different substrates, but rather to residues involved in every process that allows members of a sub-group to modify a particular subset of substrates *in vivo*. For instance, a residue that is conserved in a particular subgroup and is important for interaction with a specific partner, will have a high score in GROUPSIM, and mutation will probably result in lack of function.

In order to feed the software, we generated an alignment of alignments, comprising six yeast PATs and their orthologue groups (as defined in ORTHOMCL) making the assumption that they define specificity groups (see below). This alignment is supplied as File S1. GROUPSIM assigns each position or column in the alignment, a score which ranges from 0 to 1, and indicates how likely it is to be important for specificity determination. A table containing the scores for all the columns in the alignment and the identity of the residues for each PAT is shown in Table S2. Table 1 shows the top 11 scoring positions for the alignment, the identity of the residues present at that position for yeast Swf1 and Pfa4 and their location within protein domains.

**Table 1:** Highest scoring specificity determination positions.

Domain Name	Domain position	Groupsim score	SWF1 residue	PFA4 residue
DHHC	15	0.625792	K148	H92
DHHC	12	0.530785	A145	E89
DHHC	35	0.479647	N168	M112
TTXE	3	0.471412	N244	I200
DHHC	27	0.470615	A160	M104
DHHC	25	0.469355	L158	L102
DHHC	30	0.449352	H163	H107
DHHC	14	0.414847	S147	S91
DHHC	1	0.384384	I134	N78
DHHC	32	0.379334	I165	P109
PaCCT	2	0.375096	I322	P259

The table shows the highest scoring SDPs as determined by GROUPSIM. It also indicates the domain in which each SDP is located, the relative position within that domain and the identity of the amino acid in Swf1 and Pfa4.

Interestingly, 9 of the 11 positions correspond to residues present within the DHHC domain. Predicted SDPs outside the DHHC domain include most notably the x in TTxE motif and position 2 in the PaCCT domain as defined in González Montoro et al. (2009).

**Figure 4:**
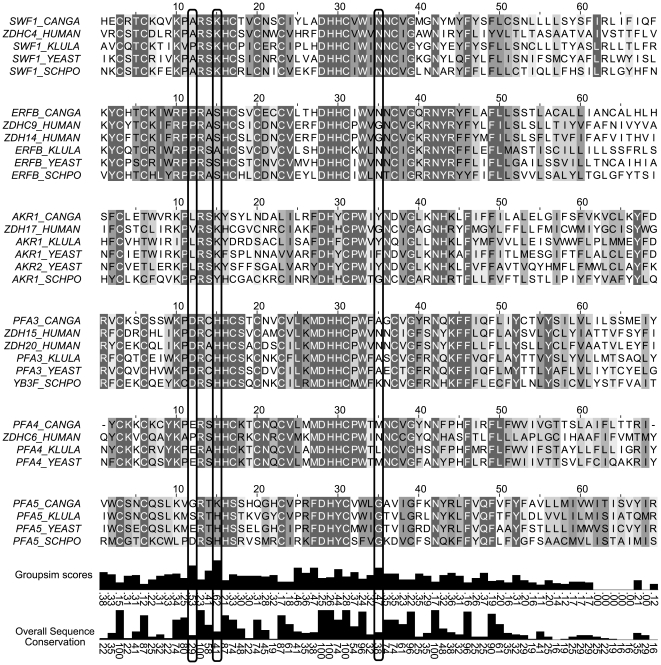
Alignment of DHHC domains from selected model organisms and Groupsim SDP prediction results. Top three highest scoring positions in the GROUPSIM analysis are boxed. Residues are shaded according to conservation within orthologue groups. GROUPSIM scores and overall conservation for each position are shown below as box plots. The more comprehensive alignment used to run the GROUPSIM analysis is shown in Figure S1.

Figure 4 displays an alignment of PATs DHHC domains from *S. cerevisiae, Homo sapiens, Candida glabrata, Kluyveromyces lactis* and *Schizosaccharomyces pombe*. Residues are shaded according to conservation within subgroups, and overall conservation is displayed in a bar graph below. GROUPSIM scores are displayed in bar graphs for each column, and the three top GROUPSIM columns are boxed, which correspond to A145, K148 and N168 in *S. cerevisiae* Swf1. The more comprehensive alignment found in File S1 was used to calculate the scores but Figure 4 can illustrate the conservation pattern of the predicted SDPs within their sequence context and also the identity of the residues for each PAT.

Some regions receive low scores in GROUPSIM analysis because they are absent from some PAT subgroups and thus cannot be aligned with the rest of the family, like the ankyrin repeat containing N-termini of Akr1 orthologous proteins. In fact, ankyrin repeats of HIP14 have been implicated in specificity determination since they can confer HIP14-like specificity to DHHC3 [28]. The N-terminal regions of Swf1 and Erf2 upstream the TMD1, as defined in [14], (which for Swf1 includes another TMD) and C-terminal regions of all PATs downstream the PaCCT motif are also excluded from the alignment.

Despite being unable to identify SDPs in unalignable regions, GROUPSIM identified several candidate SDPs, most of which are located within the DHHC domain itself.

### DHHC domains are not interchangeable

**Figure 5:**
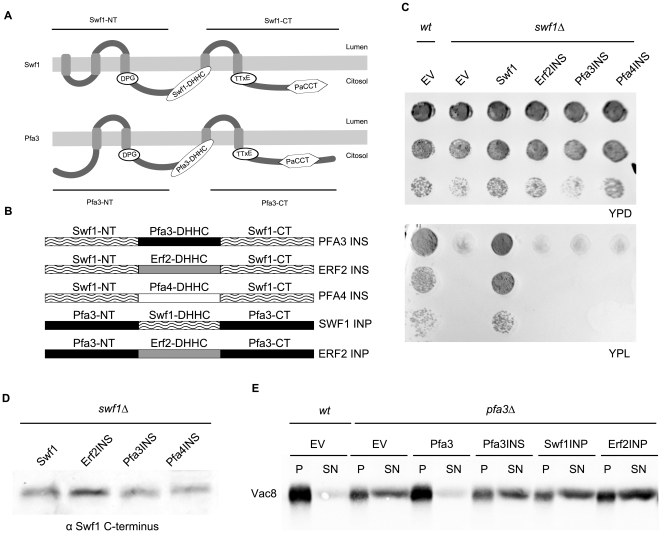
DHHC domain swapping analyses. A. Schematic representation of Swf1 and Pfa3 transmembrane topology, indicating N-terminal, DHHC and C-terminal regions used in DHHC domain swapping experiments. B. Schematic representation of the chimeric proteins used in subsequent experiments. C. Serial dilutions of a swf1Δ strain transformed with wt Swf1, or with Swf1 chimeras containing the DHHC domains from Erf2 (Erf2INS), Pfa3 (Pfa3INS) and Pfa4 (Pfa4INS). Transformants were grown in solid rich medium containing glucose (YPD, upper panel) or in medium containing lactate as the sole carbon source (YPL, lower panel). D. Western blot analysis of Swf1 and the chimeras containing the different PATs’ DHHC domains were developed using anti-Swf1 C-terminus antibodies to evaluate expression levels. E. Subcelullar fractionation assay to evaluate the membrane association of Vac8 and hence, pfa3Δ strain complementation by DHHC-swapped, chimeric genes.

It has been assumed that the DHHC domain is responsible for the catalytic activity, while substrate specificity would be encoded within the highly variable N- and C- terminal regions of PATs. Moreover, very few SDPs have been tested experimentally so, to validate our SDP prediction and the presence of SDPs within the DHHC domains, we generated chimeric genes in which we substituted the DHHC domain of Swf1 for the DHHC domains of Pfa3, Pfa4 and Erf2 (See Figure 5B for a scheme and Figure 4 for the sequences of the DHHC domains). To determine the boundaries of the DHHC domains, we selected the regions that flank the DHHC **motif**, which are highly conserved and contain almost no gaps, as shown in the alignment provided in File S1.

Topology predictions of PATs, always place the DHHC-CRD domain in a cytosolic loop between TMDs 2 and 3 defined as in Mitchel et al 2006 [14]. So a chimera in which the DHHC-CRD motif is replaced by one from a different PAT, should in principle have its transmembrane topology unaltered (Figure 5A).

The functionality of these chimeras was assayed in growth tests in medium containing lactate as sole carbon source. Figure 5C shows that none of these chimeric genes is able to restore SWF1 function. Similar results were obtained in media containing glucose and 0.85M NaCl (not shown).

To discard that lack of complementation stems from poor chimeric-protein stability, we tested the steady state levels of these proteins by Western blot, using antibodies raised against the C-terminus of Swf1, which is shared by all the constructs. Figure 5D shows that the chimeras are expressed at similar levels to that of wt Swf1. It should be noted that all these constructs are driven by the TPI1 promoter, so they are overexpressed, but still do not complement. These results suggest that, for the palmitoylation of Swf1 substrates, the DHHC domain does not simply behave as a modular catalytic unit and that perhaps specificity determinants are present within it.

Pfa3-mediated palmitoylation is less specific, since it can be carried out by other PATs, such as Erf2 [23]. We generated chimeras in which we replaced the Pfa3 DHHC domain with those of Swf1 (Swf1 INP) and Erf2 (Erf2 INP), and used these constructs in a *pfa3*Δ complementation assay.

Figure 5E shows that Vac8 soluble/membrane distribution in a *pfa3*Δ strain is not affected by expression of these chimeras. This indicates that Pfa3 DHHC domain cannot be replaced, neither by that of Swf1, which might have been expected, nor by that of Erf2, which in the context of the full length protein is able to palmitoylate Vac8. Pfa3 INS, the chimera in which Swf1 DHHC domain is replaced by that of Pfa3, is also unable to complement *pfa3*Δ. These results suggest that, besides specificity determinants that might exist within DHHC domains, particularly for Swf1, interactions between this domain and different regions of the protein are required for function or proper folding.

### Mutation of the highest scoring SDPs leads to lack of Swf1 function

**Figure 6:**
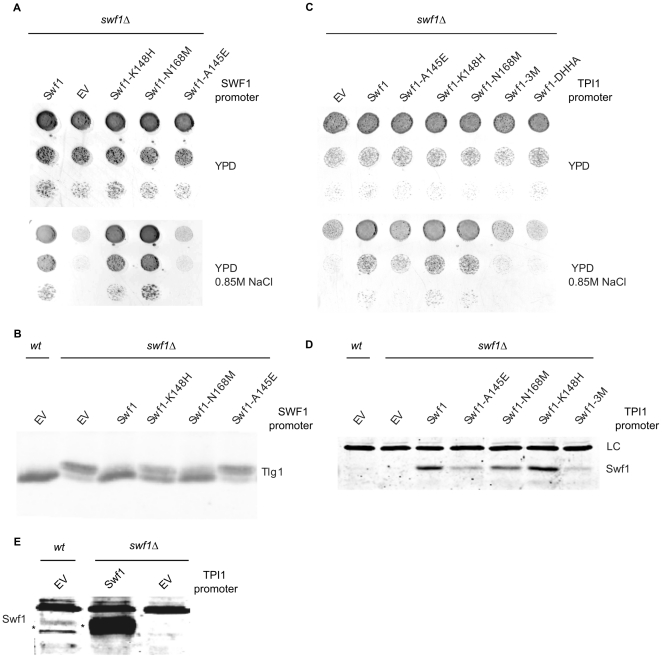
Mutation of the three top scoring SDPs in Swf1 to residues present in Pfa4. **A**. Serial dilutions of a *swf1*Δ strain transformed with an empty vector (EV), with wt SWF1, or with versions of Swf1 mutated in the highest scoring SDPs to the residues present in Pfa4 all driven by the *endogenous promoter*. Transformants were grown in solid rich medium containing glucose (YPD, upper panel) or in medium supplemented with 0.85 M NaCl (YPD 0.85 M NaCl, lower panel). **B**. Western blot of membrane proteins from a *swf1*Δ strain transformed with an empty vector (EV), wt Swf1 or swf1 alleles mutated in the three highest scoring SDPs, to equivalent residues present in Pfa4. The samples were treated with Biotin-BMCC, which biotinylates unpalmitoylated cysteines, resulting in slower migration. Blot was probed using anti Tlg1 antibodies. **C**. Serial dilutions of a *wt* strain or a *swf1*Δ strain transformed with an empty vector (EV), wt Swf1, or with versions of Swf1 mutated in the highest scoring SDPs to equivalent residues present in Pfa4, driven by the *TPI1 promoter*. Swf1-3M contains all three highest scoring SDPs mutated. Swf1-DHHA mutant is assumed to be catalytically inactive. Transformants were grown in solid rich medium containing glucose (YPD, upper panel) or in medium supplemented with 0.85 M NaCl (YPD 0.85 M NaCl, lower panel). **D**. Western blot analysis of membrane extracts from a *wt* strain transformed with an empty vector (EV) or a *swf1*Δ strain transformed with an empty vector or with TPI1 promoter driven wt or SDP Swf1 mutants. It was developed using anti Swf1 antibodies. Endogenous Swf1 is not detected under these conditions. A non-specific band is shown as a loading control (LC). **E**. Western blot analysis of a *wt* strain transformed with an empty vector (EV), and a *swf1*Δ strain transformed with an empty vector (EV) or TPI1 promoter driven Swf1. The blot was developed using anti Swf1 antibodies, and overexposed to allow for detection of endogenous Swf1.

We mutated the highest scoring positions within the DHHC domain of Swf1. Instead of a traditional alanine scanning experiment, we changed these residues to the ones corresponding to the same positions present in the DHHC domain of Pfa4, resulting in the following changes: A145E; K148H, and N168M (see Table 1). The functionality of the mutated proteins, driven by SWF1 endogenous promoter was assessed by growth tests on high salt (Figure 6A) and on lactate (not shown).

Mutation A145E renders Swf1 non-functional, indicating that this residue is indeed important for Swf1 function. Mutation K148H confers a mild growth defect and N168M shows no effect on the growth of the *swf1*Δ strain in high salt. We also analyzed the palmitoylation status of Tlg1 in *sw1f*Δ strain transformed with these SDP mutants, using the biotinylation assay. Figure 6B shows that Tlg1 is not palmitoylated by Swf1 A145E, inefficiently modified by Swf1 K148H, and palmitoylated, although not to wt levels by Swf1 N168M, in very good agreement to what is observed in the growth tests. Since we can barely detect Swf1 at endogenous expression levels, we repeated the experiment using TPI1 driven constructs, in order to analyze the stability of the mutants. In these conditions, we can still observe the lack of growth in lactate for the A145E mutant, but we are unable to see any defects for K148H. Interestingly, the triple mutant shows a more drastic growth defect compared to A145E mutant, suggesting that mutations K148H and N168M, might indeed confer an additive effect (Figure 6C). Figure 6D shows a Western blot analysis of the TPI1 driven mutant proteins, indicating that they are all expressed at high levels compared to endogenous Swf1, which in this conditions in not detected (lane 1, wt strain transformed with an empty vector). To detect endogenous Swf1, it is necessary to overexpose the blots. Figure 6E shows a comparison of endogenous Swf1 against a TPI1-promoter driven version.

The data indicates that SDP prediction can identify functionally important residues that are likely involved in the determination of specificity.

### Topological requirements for Swf1 mediated palmitoylation

**Figure 7:**
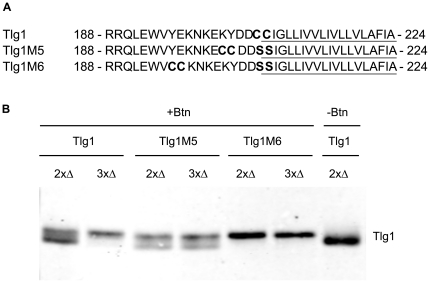
Topological requirements for Tlg1 palmitoylation. **A**. Sequences of the relevant regions from Tlg1, Tlg1M5 and Tlg1M6. The TMD region is underlined. **B**. Western blot analysis of myc-tagged Tlg1, Tlg1M5 and Tlg1M6 in *bsd2*Δ*tul1*Δ (2xΔ) or *bsd2*Δ*tul1*Δ*swf1*Δ (3xΔ) strains. Samples were treated with Biotin-BMCC, which biotinylates unpalmitoylated cysteines, resulting in slower migration. Blot was probed using anti c-myc antibodies. A non-biotinylated control sample of wt Tlg1 was included for comparison.

A logical prediction about a highly specific enzymatic reaction is that topological modification of the substrate would result in lack of recognition by the enzyme. Tlg1 has two adjacent cysteines located at the cytosolic border of the TMD. These cysteines were mutated to serines and additional cysteines were introduced at a distance of four (Tlg1M5) or ten (Tlg1M6) amino acids (Figure 7A) from their original position.

The palmitoylation status of wt and mutant versions of Tlg1 was analyzed by submitting the samples to a treatment with Biotin-BMCC, followed by SDS-PAGE and Western blot.

The experiments were carried out in *a bsd2*Δ *tul1*Δ background, as it has been established that unpalmitoylated Tlg1 is a substrate for Tul1, while versions in which cysteines are replaced for serines are substrates for degradation mediated by both these proteins [16]. In this genetic background unpalmitoylated wt Tlg1 cannot be degraded and it accumulates, and therefore a biotinylated band can be detected (Figure 7B, first lane). This amount of unpalmitoylated Tlg1 is normally not present in a wt strain [16].

Figure 7B shows that Tlg1M5 is still modified by palmitoylation, although less effectively. Interestingly, the remaining palmitoylation appears to be independent of Swf1. Tlg1M6 is no longer modified by Swf1 or any other PAT, and remains fully de-palmitoylated.

## Discussion

PATs are interesting targets for drug design since they modify oncoproteins, and this modification affects their activity. However, it would be desirable to achieve specific rather than general inhibition of palmitoylation activity. The specificity of the palmitoylation reaction is incompletely understood. It is obvious that there are many more palmitoylated substrates than enzymes, so any enzyme should be responsible for the palmitoylation of several substrates. Work in yeast seemed to indicate that each enzyme was responsible for the modification of subsets of substrates that would share certain topological features. For instance, Erf2/Erf4 seems to modify hetero-lipidated proteins, Akr1 modifies hydrophilic proteins that bind to membranes through their palmitate moieties, Pfa4 modifies multispanning membrane proteins like amino acid permeases, while Swf1 recognises single spanning membrane proteins with cysteines close to the cytosolic border of their TMDs. The need for a PAT to recognize substrates that lack a well-defined consensus sequence might result in some sacrifices in terms of specificity. Indeed, recent work by Hou et al (2009) showed that, when overexpressed, some of these proteins would modify non-canonical substrates. Moreover, deletion of Erf2 affects only 50% palmitoylation of Ras2 *in vivo* [27], and there is residual palmitoylation of Vac8 in the absence of Pfa3 [17].

In this work we analyze the specificity of Swf1 mediated palmitoylation, both from the enzyme and the substrate perspective. We provide evidence indicating that Swf1 is highly specific for its substrates since it cannot complement *pfa3*Δ or *pfa4*Δ strains, even when overexpressed. We have also shown that Tlg1 and possibly other Swf1 substrates are highly dependent on Swf1 since overexpression of other PATs cannot complement *swf1*Δ growth defects, nor the palmitoylation of Tlg1. GAL1 promoter driven Pfa3 does palmitoylate Tlg1 to some extent, but this protein is expressed at much higher level than the other PATs. Previous experiments that evaluate Tlg1 palmitoylation directly, showed almost no remaining Tlg1 palmitoylation in a *swf1*Δ mutant [16]. Roth et al. (2006), also showed that palmitoylation of Swf1 substrates was abolished by Swf1 depletion. From these experiments we conclude that, unlike Erf2, and Pfa3, Swf1 is specific and therefore an interesting model to study how palmitoylation specificity might be achieved.

A possible explanation for Swf1 specificity might be that Swf1 substrates are topologically distinct, in that cysteines are very close to the membrane, and thus this requires characteristics that are unique to Swf1.

We also carried out cross-complementation studies with Pfa4, the other PAT responsible for transmembrane protein palmitoylation. Our inability to rescue the growth phenotype in CW of a *pfa4*Δ strain even when overexpressing four different PATs, indicates that Chs3, a Pfa4 substrate, cannot be palmitoylated by other PATs. This is also supported by the work of Roth et al, (2006), in which they show that other Pfa4 substrates, such as amino acid permeases, are not palmitoylated when the PFA4 gene is deleted. It has been shown that high overexpression of Pfa4 is able to complement some Pfa3 and Akr1 deletion-associated phenotypes [23]. Therefore, Pfa4 might be more promiscuous than Swf1, however, the palmitoylation of its substrates is not. The inability of Pfa4 to complement a *swf1*Δ strain might be due to characteristics of Swf1 substrates.

Overall, we suggest that most transmembrane protein palmitoylation in yeast is highly specific. It has been reported that Sna4, an integral membrane protein, is palmitoylated by Akr1 [15]. The specificity of this reaction has not been addressed.

One aspect that should be considered in the interpretation of cross-complementation studies is the subcellular localization of the enzymes and substrates involved. Pfa4 and Erf2 are localized at the ER [27], [30]. There is some controversy regarding the localization of Swf1 [35], [16]. We have been unable to visualize endogenous Swf1 under the microscope, but when expressed from TPI1 or GAL1 promoter, it is mostly found in the ER [16, our unpublished results]. Couve et al. (1995) showed that palmitoylation of Snc1, a Swf1 substrate, occurs in the ER [36]. Pfa3 is localized at the vacuolar membrane [17]. Localization of PATs might be less relevant when analyzing peripheral membrane proteins that are able to probe all membranes until they undergo palmitoylation, this issue has been thoroughly addressed [23], [29]. For integral membrane proteins, the impossibility of co-localizing in the same membrane with a certain PAT may preclude modification, regardless of substrate-recognition mediated specificity. This could be a caveat only in Pfa3 complementation experiments of both Swf1 and Pfa4. Interestingly, when Pfa3 is highly overexpressed it modifies Tlg1 to a small degree, indicating that in principle it has access to Swf1 substrates, possibly along Pfa3 trafficking to the vacuole.

We performed extensive sequence analyses and comparison of fungal PATs. We found that there is sufficient divergence between subgroups and conservation within a subgroup to yield a few high scoring specificity determination points using GROUPSIM software. A somewhat unexpected finding is that many of these predicted specificity determinants lie within the DHHC domain, since this domain is highly conserved between all PATs studied and between different species. It should be emphasized that GROUPSIM predicts SDPs for the alignment, not just for Swf1, so some of these positions may be important specificity determinants for other PATs.

One question that arises with this prediction is just how valid it is to separate PATs in specificity groups when at least some of them have overlapping specificities. Both the uncertainty in the separation of subgroups, and the lack of experimental validation of SDP prediction [34], prompted the construction of chimeric genes in which Swf1 DHHC domain was replaced by that of Pfa3, Erf2 and Pfa4. Although the evaluation of chimeric genes requires careful interpretation, complementation of Swf1 by any one of the chimeras was plausible, and would have made us re-examine our strategy. These experiments also ruled out the simplistic view that the DHHC domain solely represents the catalytic unit, while the rest of the protein determines specificity.

SDP prediction methods require at least some degree of conservation. The ankyrin repeats present in Akr1 orthologues, the N-termini of Swf1 and Erf2 upstream TMD1 and all C-terminal regions downstream the PaCCT motif, are simply not present in the other subgroups of PATs and therefore they cannot be properly aligned. These regions are also likely responsible for specificity, or could also be involved in regulation. Indeed when a DHHC3 is fused to the ankyrin repeats of HIP14, it can modify HIP14 substrates [28].

We have mutated three high scoring positions in the DHHC domain of Swf1, to residues present in the DHHC domain of Pfa4. One of these mutations, A145E, resulted in complete lack of function, the second, K148H, resulted in an intermediate phenotype. In addition to the way in which they were predicted, an argument in favor of the involvement of Swf1 A145 and possibly K148 in specificity determination is that we have replaced them with residues that are present in an active PAT, in a sequence context of very high conservation (see Figure 4), and yet they result in lack of function. It should be noted that predicted SDPs have very low conservation scores (Figure 4) and yet mutation of at least one of them results in lack of function. On the other hand, mutations in several residues that are highly conserved across the PAT family, result in normal PAT activity (i.e C117, C123, and even D131 in Pfa3, Hou et al 2009 [23], W205 and N214 in Erf2, Mitchell et al, 2010 [37]), which suggests that SDP prediction is a useful tool to identify functionally important residues in PATs.

A more definitive proof indicating that predicted SDPs are indeed involved in specificity, would be to swap specificity of a certain PAT from one kind of substrate to another by mutating the most relevant SDPs. However, it is difficult to predict how many SDPs should be mutated, and also, the regions specific to certain PATs discussed above surely contribute significantly to specificity, making these experiments highly unlikely to succeed. We nevertheless attempted to complement a *pfa4*Δ strain using Swf1 constructs bearing the single point mutations, the triple mutant and a construct with two additional SDPs mutated to the corresponding residues in Pfa4, and neither complemented (not shown).

Finally, we analyzed the modification of Tlg1 mutants in which the cysteines have been moved away from the cytosolic border of the TMD. We show that when cysteines are moved four amino acids from their original position (see diagram in Figure 8A), palmitoylation decreases significantly. In this mutant (Tlg1M5) the sequence context of the cysteines is almost identical to wt Tlg1.

When the cysteines are moved even further, ten residues away (Tlg1M6), they are no longer palmitoylated by Swf1 or any other PAT. We cannot tell whether this is due to the distance from the membrane or a change in the amino acid context, but we can certainly state that there are constraints for the modification of these cysteines by Swf1.

Topological requirements for the palmitoylation of the H1 subunit of the asialoglycoprotein receptor have been studied in mammalian cells. Sequences surrounding the cysteines are not critical, but moving them 30 residues away from the membrane results in lack of palmitoylation [38].

It has been postulated that palmitoylation of peripheral membrane proteins occurs exclusively in the Golgi apparatus, and that the responsible machinery lacks specificity altogether, rapidly processing any available target protein. For integral membrane proteins in yeast, however, palmitoylation appears to take place at the ER [30], [36] and some of these proteins, like the SNARE Sso1, remain palmitoylated and localized to the plasma membrane without the need of a cycle through the ER [39]. In mutants where Swf1 is absent, target SNAREs move through the yeast Golgi to reach their destination but remain completely non-palmitoylated. Some mammalian transmembrane SNARES are palmitoylated [40] and it would be of great interest to study if their modification is also highly specific.

## Materials and Methods

### Ethics statement

Animal handling was performed according to the standards stated in the Guide to the Care and Use of Experimental Animals published by the Canadian Council on Animal Care and approved by the local institution (Facultad de Ciencias Químicas, Universidad Nacional de Córdoba, Argentina, Exp. N° 15-99-4042).

### Plasmids and strains

The strains used in the present study are BY4742 from the EUROSCARF consortium, or derivatives containing complete deletions of SWF1, PFA3 and PFA4. SWF1, TLG1 [16] and PFA3 [24] plasmids have been described previously. ERF2 and PFA4 coding sequences were amplified from Euroscarf BY4742 genomic DNA, using oligos erf2 01 and 02 and pfa4 01 and 02 respectively and cloned in an YCplac33 based vector containing a TPI1 promoter and PGK1 terminator (see Table S1 for a list of all oligos). For GAL1 driven constructs, PATs coding sequences were amplified using oligos that delete the stop codon and cloned in pJV95 vector as fusions to the IgG binding domain of protein A.

*bsd2*Δ *tul1*Δ strain has been described previously [41]. On this strain, SWF1 ORF was deleted by gene replacement using *S. pombe* HIS5 as a selection marker (oligos swf1 KO 5’ and swf1 KO 3′) to generate a *bsd2*Δ *tul1*Δ *swf1*Δ strain.

### Chimeric genes construction

We considered N-terminal and C-terminal regions of PATs the regions right upstream and downstream the DHHC domains defined as in Figure 6.

To generate chimeras Pfa3INS, Pfa4INS and Erf2INS, a TPI1 driven SWF1 plasmid, lacking the whole DHHC domain (pJV362) was built by PCR amplification of the N- and C- terminal coding regions. These fragments were ligated through an added X*hoI* site, using oligos swf1 01 and 42 and oligos swf1 02 and 43. The chimeras were generated *in vivo* by gap repair, using PCR amplified fragments comprising the DHHC of Pfa3 (oligos pfa3 INS 3’ and pfa3 INS 5’), Pfa4 (oligos pfa4 INS3’ and pfa4 INS 5’) or Erf2 (oligos erf2 INS3’ and erf2 INS 5’). These oligos contained aprox. 50 nucleotides of the sequences flanking Swf1 DHHC domain.

The sequence of PFA3 upstream the DHHC domain was amplified using oligos pfa3 01 and pfa3 10. This band was cloned *BamH*I-*Xho*I in pJV 362, replacing SWF1 N-terminal coding region and generating plasmid pJV368. The sequence of PFA3 downstream the DHHC domain was amplified using oligos pfa3 09 and pfa3 08. This band was cloned *Xho*I-*Pst*I in pJV368 generating plasmid pJV367. To generate chimeras in which the DHHC domain of Pfa3 was replaced, plasmid pJV367 was digested with *Xho*I and used in gap repair experiments with PCR amplified fragments comprising the DHHC domain of Swf1 (oligos swf1 INP 5’ and swf1 INP3’) or of Erf2 (oligos erf2 INP 5’ and erf2 INP 3’). A diagram of the domain structure of the chimeric proteins is shown in Figure 5A.

All constructs were rescued from yeast and verified by DNA sequencing.

### Swf1 point-mutants construction

DNA molecules corresponding to the DHHC domain of Swf1 encoding the desired mutations were purchased from GeneScript, USA. The fragments were excised from the plasmid using flanking restriction sites and used in gap repair experiments with plasmid pJV362. The constructs were rescued from yeast and verified by DNA sequencing.

### Tlg1 mutants construction

Tlg1M5 contains the following mutations: C205S; C206S; K201C and Y202C. Tlg1 M6 contains the following mutations: *C205S; C206S; Y195C and E196C*.

For TlgM5 and Tlg1M6, fragments of Tlg1 were amplified from pJV130 (Tlg1M2) [16], using oligos Tlg1 05 ad Tlg1 06 respectively and oligo M13R. Oligo Tlg1 05 replaces K201 and Y202 for cysteines and Tlg1 06 replaces Y195 and E196 for cysteines. The PCR fragments were digested with *Acc*I-*BamH*1 and cloned into a Bluescript based vector containing Tlg1 coding sequence. The mutants were then moved to a pRS316 based vector encoding a triple c-myc tag in the 5′region.

### Biotinylation assays

Biotinylation assays were performed as described in [16] but after treating with resuspension buffer, samples were diluted with sample buffer and subjected to SDS-PAGE and Western blot. Anti c-myc antibodies were from Santa Cruz and Biotin-BMCC was from PIERCE.

### Bioinformatics

*S. cerevisiae* PATs were used to query the ORTHOMCL database (release 4) [42], and 441 protein sequences corresponding to SWF1, ERF2, PFA3, PFA4, PFA5 and AKR1 orthologue groups were downloaded. MUSCLE (v3.7) [43] was used to align each group. Unalignable and poor quality sequences were removed from the dataset. PAT subgroup alignments were manually curated, and refined using UGENE (http://genome.unipro.ru) and Jalview [44]. Subsequently, these alignments were aligned using the profile-profile alignment option in MUSCLE. Final manual curation of the 303 sequences alignment was performed using Jalview. GROUPSIM [34] was used to predict specificity-determination positions using default parameters except for maximum group gap (set to 0,1).

### Anti- Swf1 antibody production and purification

A GST fusion protein of the last 100 amino acids of Swf1 (GST-Swf1CT), corresponding to the cytosolic C-terminus, was purified from *E. coli* BL21, according to the GST-fusion handbook protocol (Amersham Biosciences). 500 µg GST-Swf1CT protein in 500 µl of PBS were mixed with 500 µl of Freund’s complete adjuvant and injected intradermically into New Zealand white rabbits. Booster injections containing 125 µg of GST-Swf1CT and Freund’s incomplete adjuvant were administered 4 weeks after the initial injection. Bleeds were collected one week after each injection and screened for immuno-reactivity against Swf1 in whole cell lysates of *wt* yeast. *swf1*Δ strain was used as a negative control. Total serum was purified by adsorption to 1% ketonic powder from *swf1*Δ strain, prepared according to [45].

### Protein electrophoresis and Western blots

Protein samples for GFP-Tlg1 detection were prepared according to [46]. For the detection of the different PATs and Swf1 chimeric proteins, membrane enriched fractions were used to facilitate detection. 30 ODs of *swf1*Δ strains transformed with appropriate plasmids were harvested by centrifugation and resuspended in 300 µl lysis buffer (PBS, EDTA 2mM, PMSF 1mM and protease inhibitor cocktail (PIC). 200 µl of glass beads were added and the samples were placed in a cell disruptor (GENIE) in a cold room. The samples were submitted to four pulses of agitation of two minutes duration each, allowing one minute on ice between each pulse. 200 µl lysis buffer were added and the sample was centrifuged at 300 g for 3 min at 4°C. The supernatant was transferred to a clean tube and centrifuged at 17000 g for 20 min at 4°C. The resulting membrane enriched fraction was re-suspended in 500 µl lysis buffer plus 1% Triton X-100 and incubated on ice for 15 min. The sample was re-centrifuged at 13000 rpm for 10 min, and subjected to SDS-PAGE.

The blots were probed using secondary antibodies coupled to either IRdye 680 or IRdye 800 (LICOR bioscience, UK) at 1/20000 dilution, and then scanned using an Odyssey Infrared imager (LICOR bioscience, UK).

### Subcellular fractionation

30 ODs of yeast strains transformed with appropriate plasmids were spheroplasted with zymolyase. Spheroplasts were resuspended in 800 µl hyposmotic lysis buffer (20 mM Tris-HCl pH 7.4, 2 mM EDTA, 1 mM PMSF, PIC) (Sigma). Lysis was aided by resuspension with a small gauge needle and a syringe. The lysate was centrifuged twice for 5 minutes at 400 g at 4°C, to remove unbroken cells, and the supernatant was then centrifuged at 17000 g for 20 min at 4°C. The pellets were resuspended in 800 µl lysis buffer. Equal volumes of the pellet and supernatant fraction were subjected to SDS-PAGE and Western blot using polyclonal anti Vac8 antibodies, kindly supplied by Dr. Christian Ungermann. The intensity of the bands was quantified using the Odyssey Infrared Imager application software version 2.1.

## Supporting Information

File S1 An alignment of alignments comprising six yeast PATs and their orthologue groups (as defined in ORTHOMCL).
(TXT)

Table S1 List of oligonucleotides used troughout this work.
(DOC)

Table S2 GROUPSIM scores for all positions in the final alignment.
(XLS)
