# Supplementary material for: Specificity of Transmembrane Protein Palmitoylation in Yeast
Source: PLoS One. 2011 Feb 24;6(2):e16969. doi: 10.1371/journal.pone.0016969 (PMC3044718; doi:10.1371/journal.pone.0016969)
Supplement: Table S1 — List of oligonucleotides used troughout this work. (DOC) [file pone.0016969.s002.doc]

**Supplementary table I: List of oligonucleotides used**

| **Oligonucleotide name** | Sequence |
| --- | --- |
| calb INS 3’ | 5’-AATATGGTAAGAGTCAGAACAGCTCTAGGGAGCGTACTTGTGCTATTTAACAATGCCCAATAGCATAACC-3’ |
| calb INS 5’ | 5’-CAGGCTCGACAGAGGAATATCCATATGATTACCTATTGTATTATCCTGCCAAAACTTGTTCAACATGTC-3’ |
| calb swf1 01 | 5’-AAAGGATCCATGCTTTTTACATTAATTG-3’ |
| calb swf1 02 | 5’-TTTCTGCAGCCATTTAAGTCGTTCTATAAG-3’ |
| erf2 INP 3’ | 5’-TGAAAATCTATCAATTCACGGTTGAAACTACCGGAGTTAAACCACGTCCCAATATGGATGGCACAATTAG-3’ |
| erf2 INP 5’ | 5’-CGGAGTATATGTCAAAAAGGTGTTTGACCTTAAAACATGATGGAAGATTTAAGTATTGTCCATCATG3’ |
| erf2 INS 3’ | 5’-CGGAGTATATGTCAAAAAGGTGTTTGACCTTAAAACATGATGGAAGATTTAAGTATTGTCCATCATG3’ |
| erf2 INS 5’ | 5’-CGGAGTATATGTCAAAAAGGTGTTTGACCTTAAAACATGATGGAAGATTTAAGTATTGTCCATCATG3’ |
| pfa3 01 | 5’-CGGAGTATATGTCAAAAAGGTGTTTGACCTTAAAACATGATGGAAGATTTAAGTATTGTCCATCATG3’ |
| pfa3 02 | 5’-CGGAGTATATGTCAAAAAGGTGTTTGACCTTAAAACATGATGGAAGATTTAAGTATTGTCCATCATG3’ |
| pfa3 08 | 5’-CGGAGTATATGTCAAAAAGGTGTTTGACCTTAAAACATGATGGAAGATTTAAGTATTGTCCATCATG3’ |
| pfa3 09 | 5’-CGGAGTATATGTCAAAAAGGTGTTTGACCTTAAAACATGATGGAAGATTTAAGTATTGTCCATCATG3’ |
| pfa3 10 | 5’-CGGAGTATATGTCAAAAAGGTGTTTGACCTTAAAACATGATGGAAGATTTAAGTATTGTCCATCATG3’ |
| pfa3 INS 3’ | 5’-CGGAGTATATGTCAAAAAGGTGTTTGACCTTAAAACATGATGGAAGATTTAAGTATTGTCCATCATG3’ |
| pfa3 INS 5’ | 5’-CGGAGTATATGTCAAAAAGGTGTTTGACCTTAAAACATGATGGAAGATTTAAGTATTGTCCATCATG3’ |
| pfa4 01 | 5’-CGGAGTATATGTCAAAAAGGTGTTTGACCTTAAAACATGATGGAAGATTTAAGTATTGTCCATCATG3’ |
| pfa4 02 | 5’-CGGAGTATATGTCAAAAAGGTGTTTGACCTTAAAACATGATGGAAGATTTAAGTATTGTCCATCATG3’ |
| pfa4 INS 3’ | 5’-CGGAGTATATGTCAAAAAGGTGTTTGACCTTAAAACATGATGGAAGATTTAAGTATTGTCCATCATG3’ |
| pfa4 INS 5’ | 5’-CGGAGTATATGTCAAAAAGGTGTTTGACCTTAAAACATGATGGAAGATTTAAGTATTGTCCATCATG3’ |
| pombe INS 3’ | 5’-CGGAGTATATGTCAAAAAGGTGTTTGACCTTAAAACATGATGGAAGATTTAAGTATTGTCCATCATG3’ |
| pombe INS 5’ | 5’-CGGAGTATATGTCAAAAAGGTGTTTGACCTTAAAACATGATGGAAGATTTAAGTATTGTCCATCATG3’ |
| pswf1 01 | 5’-CGGAGTATATGTCAAAAAGGTGTTTGACCTTAAAACATGATGGAAGATTTAAGTATTGTCCATCATG3’ |
| pswf1 02 | 5’-CGGAGTATATGTCAAAAAGGTGTTTGACCTTAAAACATGATGGAAGATTTAAGTATTGTCCATCATG3’ |
| swf1 01 | 5’-CGGAGTATATGTCAAAAAGGTGTTTGACCTTAAAACATGATGGAAGATTTAAGTATTGTCCATCATG3’ |
| swf1 02 | 5’-CGGAGTATATGTCAAAAAGGTGTTTGACCTTAAAACATGATGGAAGATTTAAGTATTGTCCATCATG3’ |
| swf1 42 | 5’-CGGAGTATATGTCAAAAAGGTGTTTGACCTTAAAACATGATGGAAGATTTAAGTATTGTCCATCATG3’ |
| swf1 43 | 5’-CGGAGTATATGTCAAAAAGGTGTTTGACCTTAAAACATGATGGAAGATTTAAGTATTGTCCATCATG3’ |
| swf1 INP 3’ | 5’-CGGAGTATATGTCAAAAAGGTGTTTGACCTTAAAACATGATGGAAGATTTAAGTATTGTCCATCATG3’ |
| swf1 INP 5’ | 5’-CGGAGTATATGTCAAAAAGGTGTTTGACCTTAAAACATGATGGAAGATTTAAGTATTGTCCATCATG3’ |
| swf1 KO 3´ | 5’-CGGAGTATATGTCAAAAAGGTGTTTGACCTTAAAACATGATGGAAGATTTAAGTATTGTCCATCATG3’ |
| swf1 KO 5´ | 5’-CGGAGTATATGTCAAAAAGGTGTTTGACCTTAAAACATGATGGAAGATTTAAGTATTGTCCATCATG3’ |
| tlg1 05 | 5’-CGGAGTATATGTCAAAAAGGTGTTTGACCTTAAAACATGATGGAAGATTTAAGTATTGTCCATCATG3’ |
| tlg1 06 | 5’-CGGAGTATATGTCAAAAAGGTGTTTGACCTTAAAACATGATGGAAGATTTAAGTATTGTCCATCATG3’ |
